# Clinical Efficacy of 5-Fluorouracil and Bleomycin in Dermatology

**DOI:** 10.3390/jcm13020335

**Published:** 2024-01-06

**Authors:** Suyeon Kim, Yu Ri Woo, Sang Hyun Cho, Jeong Deuk Lee, Hei Sung Kim

**Affiliations:** Department of Dermatology, Incheon St. Mary’s Hospital, College of Medicine, The Catholic University of Korea, Seoul 06591, Republic of Korea; clarakim9511@gmail.com (S.K.); w1206@naver.com (Y.R.W.); drchos@yahoo.co.kr (S.H.C.); leejd@catholic.ac.kr (J.D.L.)

**Keywords:** 5-fluorouracil, bleomycin, dermatology, injection, topical

## Abstract

Bleomycin and 5-fluorouracil (5-FU) are widely used in various dermatological disorders. Both drugs are well-recognized as antineoplastic drugs and exert their effect by blocking the cell cycle. Topical and intralesional formulations are available and have been studied in both non-neoplastic and cancerous lesions. However, data comparing the effect of bleomycin and 5-FU in the dermatological disorders are limited. This review outlines the action mechanisms of both drugs and compares their clinical efficacies in a wide range of dermatologic diseases including hypertrophic scar, wart, skin cancer, vascular malformation, hemangioma, and vitiligo, and discusses the overall safety of the drugs. Intralesional bleomycin treatment is effective in hypertrophic scars and warts, but intralesional 5-FU may also be considered since it is cheaper and less painful. Moreover, intralesional 5-FU and bleomycin injection is a viable option for premalignant lesions (i.e., actinic keratosis) and inoperable skin cancers. Both bleomycin and 5-FU have been applied as treatment adjuncts for vitiligo, with 5-FU showing a slightly better outcome. Both agents have a good safety profile, and no serious side effects have been reported following their use in the field of dermatology.

## 1. Introduction

Bleomycin is an antitumor antibiotic which effectively blocks the cell cycle and cleaves DNA [1]. Bleomycin is composed of water-soluble glycosylated peptides, a key factor in its action mechanism. By chelating Fe (II) (Fe^2+^, element iron in its +2 oxidation state) to Fe (III) (Fe^3+^, element iron in its +3 oxidation state) and creating free radicals, bleomycin exerts oxidative damage to the DNA. In addition to solid tumors and Hodgkin’s lymphoma, the FDA has approved systemic bleomycin as an adjunct chemotherapy agent for squamous cell carcinoma on the head and neck, cervix, penis, and skin [2]. 

5-Fluorouracil (5-FU) is a pyrimidine analog that is composed of a pyrimidine and furan ring. 5-FU can be incorporated into both DNA and RNA and disrupts their synthesis by inhibiting thymidylate synthase [3]. 5-FU, a chemotherapeutic agent, is well recognized for its use in many cancer types including ovarian, gastroenterology, and breast cancer. In the dermatology field, the FDA has approved topical 5-FU as a treatment modality for actinic keratosis and superficial basal cell carcinoma [4]. 

Dermatologists have been actively using bleomycin and 5-FU off-label for various skin diseases such as warts, hypertrophic scars, vascular malformations, and cutaneous malignancies. Currently, bleomycin is available as lyophilized bleomycin sulfate powder in a vial (15 U/vial), and 5-FU is available at a concentration of 50 mg/mL and in a topical form. Electroporation, micro-needling, and local anesthetics can be combined with bleomycin or 5-FU to improve cell permeabilization and relieve pain. Because of the similar action mechanisms, their usage in skin diseases overlaps. This review discusses the general features of the two drugs and compares their indications, side effects, and efficacy. 

## 2. Materials and Methods

This literature review was conducted after searching PubMed for articles published between 1 January 1980, and 31 December 2023, and identifying the usage of bleomycin and 5-FU in dermatology and comparing their effects. The following terms were used in combinations: *“5-Fluorouracil”*, *“5-FU”*, *“Bleomycin”*, *“dermatology”*, *“intralesional”*, and *“topical”*. Further searches were conducted regarding safety and the common indications for 5-FU and bleomycin treatment, including *hypertrophic scar*, *wart*, *skin cancer*, *vascular malformation*, *hemangioma*, *and vitiligo*. 

Studies and reviews examining the use of bleomycin and 5-FU in the field of dermatology as a single treatment or in combination with other therapeutic options were included. The exclusion criteria are as follows: studies which lack objective data on the efficacy of bleomycin or 5-FU, reports of combined therapy with outdated or experimental treatment modalities, and studies in which the primary focus was not dermatologic indications.

## 3. Results

Overall, 47 studies were included: 7 studies for hypertrophic scar and keloid, 11 studies on wart, 18 studies in skin cancer, 4 studies for vitiligo, 2 studies applied to hemangioma, and 7 studies on other indications.

This review outlines the action mechanisms of both drugs and compares their clinical efficacies in a wide range of dermatologic diseases including hypertrophic scar, wart, skin cancer, vascular malformation, hemangioma, vitiligo, and various other diseases (Table 1).

## 4. Administration

In dermatologic practice settings, bleomycin is directly injected into the lesion, whereas 5-FU can be applied topically or delivered through injection [5,6]. Since bleomycin is a hydrophilic drug, its topical application can be disappointing in terms of drug delivery to the target site. Electroporation, micro-needling, and ablative fractional lasers can improve the trans-epidermal absorption of bleomycin by disrupting the skin barrier, allowing bleomycin to be better taken up into the target cells [7]. Bleomycin is available as lyophilized bleomycin sulfate powder in a vial (15 U/vial), and for cutaneous injection, dilution with 15 mL of normal saline (dilution 1 mg/mL) is recommended. Higher concentrations of bleomycin can increase the risk of skin necrosis and ulceration.

5-FU is available at a concentration of 50 mg/mL and is usually injected without further dilution. Its topical form is prepared in numerous concentrations (0.5%, 1%, 4%, and 5% for cream, and 2% and 5% for solutions), and 0.5% cream is most widely used since it causes the least irritation, with maximum patient compliance. 

## 5. Safety

### 5.1. Bleomycin

The best-known and most serious side effect of systemic bleomycin is pulmonary fibrosis. Fever, vomiting, loss of appetite, and a sore throat can also be induced. The side effects strongly depend on the dosage. Severe toxicities occurred with total doses greater than 150 mg in an intravenous route [1]. There are no known antidotes. However, no systemic adverse effects have been reported after intralesional bleomycin injection for dermatologic indications, which mostly do not exceed 5 mg in total [1]. 

The most common side effects of local injections are pain and hyperpigmentation. Local pain can be more severe than other injections because of its sclerosing effect. Local anesthesia before an injection is usually sufficient to alleviate pain. New methods including the micropuncture method, microneedling pen, laser-assisted drug delivery, and needle-free pneumatic injection have been applied to avoid injection pain [1]. The injection site redness, swelling, and burning sensation are usually transient. 

Eschar formation and blackening of the skin can commonly occur. These reactions are mostly the result of effective treatment but need to be checked if there is any sign of an ulcer. A hematoma can develop if the injection goes into the deep dermis. Raynaud’s phenomenon, onychodystrophy, scleroderma, flagellate erythema, and gangrene are rarely reported [2]. The contraindications of bleomycin include pregnancy (Category D), allergic reaction, Raynaud’s phenomenon, and peripheral vascular diseases [1].

### 5.2. 5-FU

The most serious side effects of systemic 5-FU are myelosuppression and mucositis. Other common antitumor drug side effects can be easily induced. However, like bleomycin, no serious systemic adverse effects have been reported following the local use of 5-FU in a range of dermatologic indications. 

Local irritation and pain are common side effects of 5-FU. Hyperpigmentation, ulceration, and inflammatory reactions are also reported, but most are transient [3]. Although similar side effects have been reported with both topical and intralesional 5-FU, intralesional 5-FU is more likely to be associated with pain, hyperpigmentation, and blisters. Furthermore, superficial necrosis, local infection, and wound dehiscence are rarely reported as serious complications.

Topical 5-FU is widely accepted for its effects on many dermatologic indications, and therefore topical agents can be considered as alternatives to intralesional agents if the patient is intolerant to a burning sensation. In most cases, injection pain can be controlled with local anesthesia and cold air.

In general, both bleomycin and 5-FU injection can cause pain, but in real experience, including in our institution, patients complain more about pain with bleomycin injections. This is likely due to their sclerosing effect which causes more tissue irritation. In addition, since bleomycin is in the acidic range (pH 3–4), in contrast with 5-FU, which has a neutral pH (pH 7–8), this can be another factor that causes more tissue destruction. 

## 6. Hypertrophic Scar and Keloid

### 6.1. Efficacy

Hypertrophic scar and keloid are both abnormal tissue reactions following inflammation, trauma, or surgery. While a regular wound healing process recruits inflammatory cells, keratinocytes, and fibroblasts, its excessive and prolonged activation can lead to the formation of hypertrophic scars and keloids. 

Since bleomycin induces the apoptosis of keratinocytes and inhibits collagen synthesis, it has also been applied to hypertrophic scars and keloids via intralesional administration. A meta-analysis study on the efficacy of bleomycin identified that bleomycin had a greater effect than other treatments, including triamcinolone (TAC) and 5-FU [8]. 

5-FU also exerts its effect on scars through its antimetabolic activity and the suppression of fibroblast proliferation [9]. Three randomized controlled trials (RCTs) reported 5-FU to be comparable to TAC [3,10]. In addition, one RCT and one comparative study showed that the combination of intralesional TAC with 5-FU was superior to 5-FU alone [11,12]. 

### 6.2. Dosage and Techniques

As for bleomycin, intradermal injection and the multipuncture method are effective [2]. Bleomycin is most often diluted with normal saline or distilled water to a concentration of 1.0 to 1.5 mg/mL per dosage [1]. A combination with intralesional TAC can be a better option on refractory keloid than using bleomycin alone (0.3 mL of diluted bleomycin mixed with 0.3 mL of TAC 40 mg/mL and 0.3 mL of lidocaine) [13].

For 5-FU, it is manufactured at a concentration of 50 mg/mL and 0.2–0.4 mL is usually injected per/cm^2^ area [2,10]. A combination with intralesional TAC can be a better treatment option than using 5-FU alone (0.9 mL of 50 mg/mL 5-FU mixed with 0.1 mL of TAC 40 mg/mL) [11,12].

**Figure 1:**
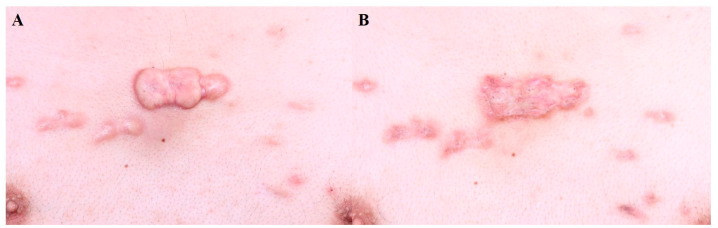
Truncal keloid, (**A**) before treatment and (**B**) after 7 sessions of bleomycin injection (every 4 weeks).

A comparative study showed that intralesional bleomycin resulted in greater improvements in hypertrophic scars than intralesional 5-FU injection [14]. The depth of 5-FU and bleomycin injection should be aimed at the mid-dermis to avoid necrosis or ulceration [1]. Injections at 2–4-week intervals are recommended. Figure 1 demonstrates the treatment of a truncal keloid with seven sessions of bleomycin injection at 3–4-week intervals. A final follow-up was performed after six months with no sign of recurrence. 

## 7. Wart

### 7.1. Efficacy

Wart is an abnormal epidermal proliferation caused by human papillomavirus (HPV) infection in keratinocytes. Many topical and intralesional agents have been studied for the treatment of warts, since cryotherapy alone is often not satisfactory in terms of efficacy. 

Since bleomycin blocks the cell cycle and cleaves DNA in viruses, it has been applied to warts since the 1970s [1]. Two RCTs reported that intralesional bleomycin had significantly higher cure rates than cryotherapy [15,16]. In addition, it had superior effects on recalcitrant warts [16,17]. A comparative study showed that intralesional bleomycin injection had higher clearance rates of warts compared to 5-FU and Candida albicans antigen injection [18].

5-FU exerts its effects on warts by disrupting viral DNA synthesis. Two RCTs evaluated the efficacy of intralesional 5-FU and local anesthesia (4 mL of 50 mg/mL 5-FU, 1 mL of a mixture of 20 mg/mL lidocaine, and 0.0125 mg/mL epinephrine), comparing it with saline injection with the successful treatment of 65% of the warts [19,20]. However, an RCT reported that combining topical 5-FU with cryotherapy had no additional benefit when compared to cryotherapy alone [21]. 

### 7.2. Dosages and Techniques

Bleomycin is usually diluted with normal saline or distilled water at a concentration of 0.5 or 1.0 U/mL [17]. Typically, 0.2 mL is injected into 5 mm warts and up to 1 mL in larger warts [15]. Intralesional injection is the most widely chosen administration technique, but the multipuncture method or a microneedling pen can be applied to reduce pain [22]. According to a prospective study, a local electroporation procedure after intralesional bleomycin injection showed a higher cure rate than bleomycin alone [23]. 

For 5-FU, intralesional injection (50 mg/mL) into the wart is often recommended. Two RCTs showed that topical 5-FU under occlusion or in combination with salicylic acid could enhance the effect of 5-FU cream [24,25]. 

**Figure 2:**
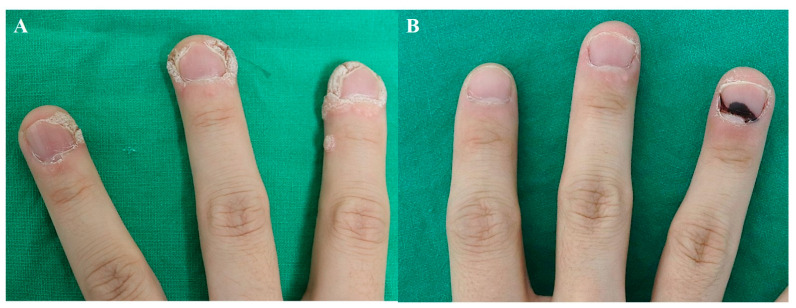
Periungual warts, (**A**) before treatment and (**B**) after 2 sessions of bleomycin injection (2 weeks apart).

Unlike hypertrophic scars, the injections on warts should be aimed at the superficial dermis, since epidermal necrosis is important for eliminating the wart [1]. Injections at 2–4-week intervals are recommended. Figure 2 demonstrates the disappearance of periungual warts following two sessions of bleomycin injection at an interval of 2 weeks. 

## 8. Skin Cancer

### 8.1. Efficacy

Complete excision remains the mainstay of treatment for skin cancer [26]. However, for patients who are not optimal candidates for surgery due to old age or co-morbidities, local bleomycin or 5-FU can be a viable option. Since bleomycin and 5-FU block the cell cycle and disrupt DNA synthesis, trials on skin cancer have been conducted. Overall, data are limited on the clinical efficacy of bleomycin and 5-FU on skin cancer and only 5% 5-FU cream is FDA-approved for the treatment of actinic keratosis and superficial basal cell carcinoma [26]. In this review, studies that examined the efficacy of bleomycin and 5-FU on actinic keratosis (AK), basal cell carcinoma (BCC), squamous cell carcinoma (SCC), keratoacanthoma (KA), and metastatic melanoma are included.

### 8.2. Actinic Keratosis

Topical 5-FU has been widely used to treat AK with sufficient clinical data on its efficacy [27]. Two RCTs identified that combining topical 5-FU with cryotherapy resulted in a higher clearance of AK than cryotherapy alone [28,29]. The study also recommended the once-daily application of a 0.5% formulation, as it offers similar efficacy to 5% 5-FU cream while causing less irritation, thereby increasing patient compliance [29]. In countries where 5-FU cream is not available, and in cases of full-thickness irregularity (Bowen’s disease) and fewer lesions, intralesional 5-FU can be attempted.

### 8.3. Basal Cell Carcinoma

The FDA has approved 5% 5-FU cream for superficial BCC and has numerous data to support its effect [26,30]. However, there are few trials and case studies on the use of intralesional 5-FU on BCC. Miller et al. (2009) [30] conducted an RCT using an investigational agent (i.e., intralesional 5-FU (30 mg/mL)/epinephrine (0.1 mg/mL) gel) for superficial and nodular BCC. Although this gel formulation is not yet commercially available, the results were promising.

There have been two clinical trials on bleomycin-mediated electrochemotherapy (ECT) for BCC [31,32] where electroporation was used to increase the penetration of bleomycin into cancer cells. Glass et al. (1997) [31] laid out a protocol for injecting 0.5 to 1 mg of bleomycin depending on the tumor size, followed by the delivery of an electric pulse. In this study, most patients (94%) required only a single session of ECT. The second ECT study offered treatment every 3 weeks until complete remission was achieved, with the mean follow-up period being 8.6 months [32]. Recently, the intralesional delivery of bleomycin via micro-infusion (MMP^®^) was introduced and was found to be effective in BCC [33]. There is a case report in which BCC was cleared with intralesional bleomycin injection alone [34].

### 8.4. Squamous Cell Carcinoma and Keratoacanthoma

Topical 5-FU (5%) has been applied to Bowen’s disease (SCC in situ) [35,36]. However, more research is needed since the data are scarce and outdated.

The use of intralesional 5-FU has been widely studied for SCC and KA [37]. One study involving 172 SCC lesions showed a high cure rate of SCC with intralesional 5-FU injection, where 158 (92%) showed clinical resolution after treatment [38]. In a case series of 20 SCC patients, intralesional 5-FU injection was said to remove the tumor in 19 patients (95%) without any recurrence [39]. In addition, the successful use of 5-FU for SCC on cosmetically sensitive areas has been reported [40,41]. Therefore, intralesional 5-FU may be considered a promising alternative to surgery in patients with KAs, and even for invasive SCC on cosmetically sensitive areas.

Since there are few reports of KA and SCC treated with intralesional bleomycin, further studies are needed to confirm its efficacy. Considering that bleomycin has a stronger effect than 5-FU, it is expected to provide more positive results.

### 8.5. Metastatic Melanoma

5-FU and bleomycin are rarely applied to primary melanoma. However, they may be applied to control cutaneous metastasis and for palliative treatment. Intralesional bleomycin ECT has shown an effect in controlling metastatic melanoma where intralesional bleomycin ECT was superior to intralesional bleomycin alone [42,43].

### 8.6. Dosages and Techniques

The dose and number of treatments vary depending on the tumor size. Generally, 0.5 to 2 mL injections at an interval of 1 week until complete clearance is recommended [26]. Here, complete resolution is claimed when no tumor cells are found in a follow-up biopsy. The concentration of bleomycin ranged from 0.5 to 1.5 U/mL, whereas that of 5-FU was fixed at 50 mg/mL [26]. Bleomycin-mediated ECT was the most recommended [42]. 

**Figure 3:**
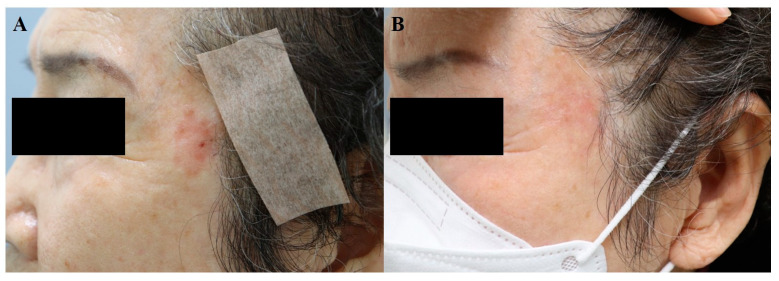
Bowen’s disease, (**A**) before treatment and (**B**) after 8 sessions of 5-FU injection (at an interval of 2–3 weeks).

In summary, although surgery remains the treatment of choice for skin cancer, the intralesional injection of 5-FU or bleomycin can be considered in special circumstances (i.e., old age, debilitating condition) for BCC, SCC, and melanoma skin metastasis, under close supervision. Figure 3 demonstrates the regression of Bowen’s disease on a cosmetically sensitive area in an 81-year-old woman. After eight sessions of 5-FU injection, each spaced 2–3-weeks apart, a follow-up biopsy confirmed the disappearance of atypical cells.

## 9. Vitiligo

### 9.1. Efficacy

Vitiligo is an autoimmune disease mediated by T cells which results in the destruction of melanocytes and skin depigmentation. Treatment is based on the extent and activity of the disease, with phototherapy and topical agents (i.e., topical calcineurin inhibitors, topical steroids) being the mainstay of therapy.

Topical 5-FU was first introduced for the treatment of vitiligo by Tsuki and Hamada (1983) [44]. 5-FU induces the proliferation of melanocytes in the hair follicles, which migrate to the epidermis and produce melanin [45]. Many acknowledge that superficial wounding via dermabrasion or fractional CO_2_ or erbium: YAG laser can enhance the penetration of 5-FU cream. One RCT suggested 5-FU with microneedling to have higher efficacy compared to 5-FU cream alone in vitiligo (*p* = 0.03) [45]. This study included patients only with stable vitiligo and the regimen required they apply topical 5% 5-FU twice daily for 14 days. Another RCT suggested that topical 5-FU following erbium: YAG (2940 nm) laser is more effective than 5-FU cream alone [46]. Instead of applying the 5-FU cream, the 5-FU solution (50 mg/mL) can be sprayed on the superficial wounds.

Intralesional 5-FU injection is another option for vitiligo. One RCT suggested that 5-FU injection was more effective than intralesional triamcinolone (*p* = 0.025 in the face, *p* = 0.043 in acral lesions) [47]. In this study, patients with stable vitiligo were recruited, and 0.1–0.2 mL of 5-FU (50 mg/mL) was injected per point, spaced 1 cm apart. A total of four intradermal injections were performed at an interval of 2 weeks.

### 9.2. Dosage and Techniques

**Figure 4:**
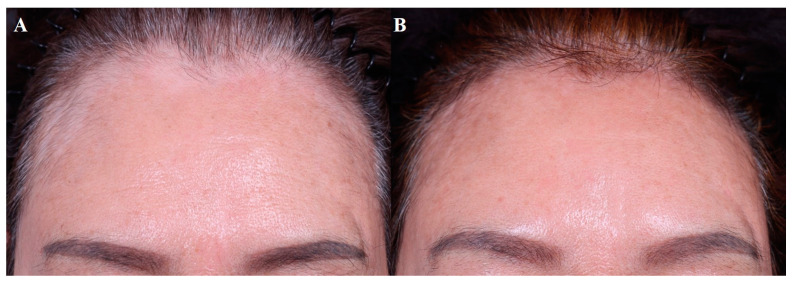
Facial vitiligo, (**A**) before treatment and (**B**) after 4 sessions combined treatment with CO_2_ fractional laser and bleomycin (each performed every 4–8 weeks).

Most studies included patients with at least one year of stable disease. Topical 5-FU combined with dermabrasion or laser treatment is a widely used technique, but intralesional 5-FU can be another option. Once-daily application is recommended for 5-FU cream, whereas the intralesional injection of 5-FU is performed every 2 weeks. Studies of vitiligo treated with intralesional bleomycin are lacking but may be a viable option. Figure 4 demonstrates an improvement of facial vitiligo after four sessions of CO_2_ fractional laser plus bleomycin, where bleomycin solution was sprayed on the wounded site every 4–8 weeks.

## 10. Vascular Anomalies

### 10.1. Efficacy

In accordance with the International Society for the Study of Vascular Anomalies (ISSVA) classification updated in 2014, vascular anomalies can be divided into vascular malformations and vascular tumors. Infantile hemangioma and congenital hemangioma are included in vascular tumor and vascular malformations and are subdivided into capillary, lymphatic, venous, and arterial malformations. Hemangioma is the result of endothelial hyperplasia, while vascular malformation is the result of a vascular system error during embryonic development.

Intralesional bleomycin has been used in these diseases, with its sclerosing and antineoplastic effect, which induces apoptosis in immature proliferating cells [2].

### 10.2. Hemangioma

As hemangiomas often leave scars, early intervention is becoming increasingly popular over the ‘wait and see’ approach. While vascular lasers and oral propranolol are the first-line treatment options for hemangioma, intralesional bleomycin is applied to patients who show resistance and in individuals who would benefit from additional treatment. One RCT suggested that intralesional bleomycin was more effective than triamcinolone by 87.5% (excellent response + good response) (*p* = 0.037) in reducing the size of the hemangioma [48].

### 10.3. Vascular Malformation

Although surgery and ethanol injection are the preferred options for vascular malformation, diffuse microcystic malformations can benefit from bleomycin injection. A systematic review identified bleomycin sclerotherapy to be effective in lymphatic malformations (84%) and venous malformations (87%) [49]. While bleomycin is comparable to other sclerosants in terms of efficacy, it has a far better safety profile, which makes it a suitable treatment option. The size of a vascular malformation can be assessed clinically or combined with radiologic images including Doppler ultrasonography and MRI [49]. 

### 10.4. Dosage and Techniques

**Figure 5:**
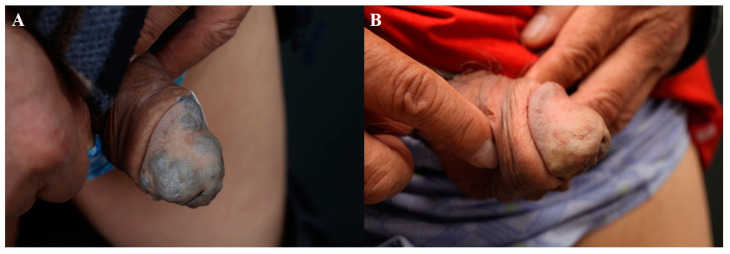
Venous malformation, (**A**) before treatment and (**B**) after 3 sessions of bleomycin injection (spaced 3–4 weeks apart).

Intralesional bleomycin is a popular adjunct treatment modality. Since individuals who come in with vascular anomalies are mostly babies or children of a young age, most are prone to skin ulceration. Accordingly, small doses of bleomycin prepared in relatively low concentrations (0.5–1 mg/mL) are recommended. The maximum dose is usually set at 0.5–1 mg/kg for children under one year of age and 15 mg for those who are older. After the injection, the lesion should be compressed for at least 10 min. Normally, 4–10 sessions of bleomycin injections are given, each 2–4 weeks apart [2]. Figure 5 demonstrates the improvement of a venous malformation following 3 sessions of bleomycin injection spaced 3–4 weeks apart (Reprinted from [50]).

## 11. Other Indications

**Figure 6:**
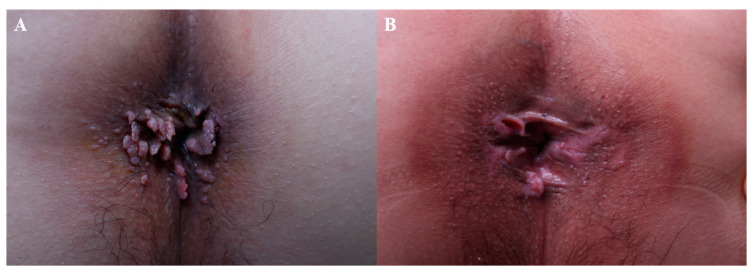
Condyloma acuminatum, (**A**) before treatment and (**B**) after 2 sessions of bleomycin injection (spaced 2 weeks apart).

Bleomycin and 5-FU have been studied in miscellaneous skin diseases. In the case of corns and calluses, a comparative study showed that intralesional bleomycin had an added benefit to paring alone [51]. In this study, 1 mL of bleomycin (1 mg/mL) or less was injected into the lesion. Injections with maximum of 10 sessions were performed with intervals of 3 weeks. A case series showed the resolution of condyloma acuminatum after administering bleomycin every 2 to 3 weeks [52]. Figure 6 demonstrates the clearance of multiple perianal condyloma acuminata following two sessions of bleomycin injection spaced 2 weeks apart. There was no sign of recurrence at the final follow-up, made after six months.

**Figure 7:**
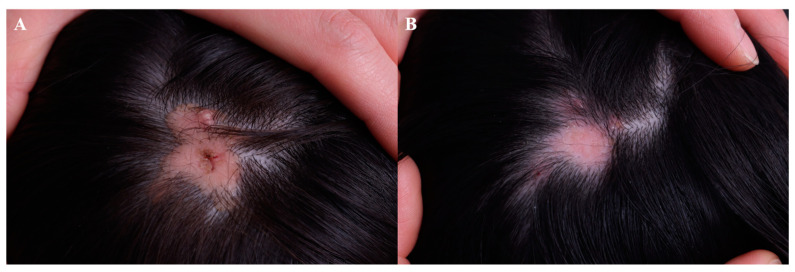
Nevus sebaceous, (**A**) before treatment and (**B**) following 4 sessions of 5-FU injection after a primary erbium: YAG and Co_2_ laser ablation (5-FU injection given every 4–8 weeks).

A patient with cutaneous sarcoidosis was successfully managed with intralesional 5-FU [53]. In addition, intralesional triamcinolone combined with 5-FU (0.1 mL of 40 mg/mL TAC plus 0.9 mL of 50 mg/mL 5-FU) may be applied to refractory inflammatory nodules including filler granuloma [54]. Dysplastic nevus was successfully managed with topical 5-FU according to a case series involving six patients [55]. Inflammatory linear verrucous epidermal nevus reportedly improved following a combined regimen of 0.1% topical tretinoin and 5% 5-FU cream [56,57]. Recently, intralesional 5-FU was shown to work on refractory cutaneous T-cell lymphoma in combination with imiquimod cream [58]. Figure 7 demonstrates the regression of nevus sebaceous following four sessions of 5-FU injection after a primary erbium: YAG and CO_2_ laser ablation, each spaced 4–8 weeks apart. A final follow-up was made after 1 year with no sign of active proliferation.

## 12. Conclusions

In summary, bleomycin and 5-FU are a promising treatment option for many skin diseases including hypertrophic scars, warts, non-melanoma skin cancers, vitiligo, and vascular anomalies, with a slightly superior effect for bleomycin compared to 5-FU. Intralesional bleomycin treatment led to great improvements in hypertrophic scars and warts, but intralesional 5-FU may also be considered to be a treatment option since it is cheaper and less painful. Based on the current data, local 5-FU and bleomycin can be considered as successful adjuncts or alternatives to surgery for malignancies including BCC, SCC, AK, and vitiligo. Along with intralesional triamcinolone and botulinum toxin injections, tiny doses of intralesional bleomycin may be promising in scar prevention after operations. Intralesional bleomycin also works effectively when combined with vascular lasers or beta blockers for infantile hemangiomas. Further research regarding intralesional bleomycin on skin cancer is needed, with the data being both scarce and outdated.

Although limited to case reports, intralesional bleomycin and 5-FU have been applied to challenging conditions such as filler granuloma or refractory cutaneous T-cell lymphoma, which suggests the potential for future use, with space for additional research.

Both bleomycin and 5-FU have a good safety profile when applied locally. The most common side effect associated with intralesional bleomycin and 5-FU injection is pain during and after treatment. Patients would benefit from future research focusing on pain reduction.

## Figures and Tables

**Table 1:** Uses of bleomycin and 5-FU ^1^ in dermatology.

Indications	Mechanisms of Action	Bleomycin (Injection 0.5–1.0 U/mL)	5-FU (Topical or Injection 50 mg/mL)	Recommended Interval
Hypertrophic scar	Induces apoptosis of keratinocytes and inhibits collagen synthesis	Intralesionally into the mid-dermis		2–4 weeks
Wart	Blocks the cell cycle and cleaves DNA in viruses	Intralesionally into the superficial dermis		2–4 weeks
Skin cancer	Blocks the cell cycle and disrupts DNA synthesis	For patients who are unable to undergo surgery		Once daily for topicals, spaced 1–2 weeks apart for injection
Actinic keratosis		*Scarce data*	Topical 5-FU with cryotherapy	
Basal cell carcinoma		0.5 to 1.5 U/mL Bleomycin ECT is recommended	Topical 5-FU (*limited to superficial BCC* ^2^)	
Squamous cell carcinoma		*Scarce data*	Intralesional 5-FU	
Metastatic melanoma		0.5 to 1.5 U/mL bleomycin ECT is recommended	*Scarce data*	
Vitiligo	5-FU: induces the proliferation of melanocytes in hair follicles	*Scarce data*	For patients with stable disease, topical agents following dermabrasion or intralesional injection	Once daily for topicals, space 2 weeks apart for injection
Vascular anomalies	Bleomycin: its sclerosing and antineoplastic effect, which induces apoptosis of the immature cells	0.5 mg/mL 0.5–1 mg/kg (under 1 year) Maximum 15 mg (aged 1 and ove)		2–4 weeks
Hemangioma		For patients resistant to propranolol and vascular laser	*Scarce data*	
Vascular malformation		For lymphatic and venous malformation		
Other indications		Corn/Callus Condyloma acuminatum	Cutaneous sarcoidosis Refractory inflammatory nodule Dysplastic nevus Cutaneous T-cell lymphoma	

^1^ 5-fluorouracil. ^2^ Basal cell carcinoma.

## Data Availability

The data that support the findings of this study are available on request from the corresponding author.
